# Contextual influences affecting patterns of overweight and obesity among university students: a 50 universities population-based study in China

**DOI:** 10.1186/s12942-017-0092-x

**Published:** 2017-05-08

**Authors:** Tingzhong Yang, Lingwei Yu, Ross Barnett, Shuhan Jiang, Sihui Peng, Yafeng Fan, Lu Li

**Affiliations:** 1 Department of Social Medicine/Center for Tobacco Control Research, Zhejiang University School of Medicine, Hangzhou, 310058 China; 2 Department of Geography, University of Canterbury, Private Bag 4800, Christchurch, New Zealand; 3 Department of Nursing, Hangzhou Normal University, Hangzhou, 310036 China; 4 Institute of Family and Social Medicine, Zhejiang University School of Medicine, Hangzhou, 310058 China

**Keywords:** Overweight and obesity, Young adults, Context effects, China

## Abstract

**Background:**

Many studies have examined childhood and adolescent obesity, but few have examined young adults and the effect of their home and current living environments on prevalence rates. The present study explores contextual factors affecting overweight and obesity among university students in China and, in particular, focuses on how the SES–obesity relationship varies across different geographical contexts.

**Methods:**

Participants were 11,673 students, who were identified through a multistage survey sampling process conducted in 50 universities. Individual data was obtained through a self-administered questionnaire, and contextual variables were retrieved from a national database. Multilevel logistic regression models were used to examine urban and regional variations in overweight and obesity.

**Results:**

Overall the prevalence of overweight and obesity in the study sample was 9.5% (95% CI 7.7, 11.3%). After controlling for individual factors, both attributes of the home location (regional GDP <gross domestic product> per capita and rurality) and the current university location (city population) were found to be important, thus suggesting that the different origins of students affect current levels of obesity. At the individual level, while students with more financial resources were more likely to be obese, the extent of this relationship was highly dependent upon area income and city size.

**Conclusion:**

The results of this study add important insights about the role of contextual factors affecting overweight and obesity among young adults and indicate a need to take into account both past as well as present environmental influences when considering the role of contextual factors in models of the nutrition transition.

## Background

The prevalence of overweight and obesity is increasingly evident in both richer and poorer countries [1]. Although greater attention has been paid to environmental determinants of obesity in recent years [2], this research has largely occurred in western countries and focused on various neighborhood factors affecting obesity prevalence [3]. In low and middle income countries, while the impact of the nutrition revolution has been noticed for some time [4, 5], less attention has been paid to the independent effect of contextual risk factors affecting overweight and obesity and how these may differ from those of richer countries. However, the studies which have occurred have largely focused on factors affecting national variations in obesity [6] and variations in the impact of socio-economic status (SES) [6] and other contributing factors [7]. While others have explored urban and regional differences in obesity at the sub-national scale [8], many of these studies have not always adequately controlled for individual level factors [9]. In some cases regional variations in obesity are largely seen as an outcome of individual level differences [10] or where independent macro-level effects have been identified, with few exceptions [11, 12], often these have been unspecified [13, 14]. Thus, in view of such trends, it is important to pay greater attention to the significance of various environmental determinants of obesity and why these may be important in countries at earlier stages in the nutrition revolution. The objective of this study, therefore, is to investigate the effects of both the home (region) and current (university city) living contexts on obesity among university students in China.

Because of their high mobility rates, studies of obesity among young adults provide an opportunity to simultaneously examine the effect of a variety of contextual effects, characteristic of their home and current locations, which may contribute to obesity [15]. Current patterns of obesity most likely reflect different cultural and behavioral norms relating to the home locations of students [16] as well as the socio-economic and other characteristics of the environments where they now reside. A frequent criticism of contextual studies of health is that they are cross-sectional in nature and do not take account of prior environmental conditions that people have been exposed to. While there have been many studies of childhood and adolescent obesity [17], there has been less focus on the importance of earlier life conditions on current levels of obesity. For example, in the United States, Zheng and Tumin [18] found that women’s obesity status at older ages was influenced by early childhood conditions and place of residence, while adulthood factors seemed to be more important for males. Among the few studies of younger adults the evidence suggests that, for some groups (e.g. African Americans) neighborhood deprivation clearly plays a role in later patterns of obesity [19]. Similarly in Denmark, birthplace played a role in explaining regional differences in the prevalence of obesity. Young men currently living in provincial rural areas surrounding Copenhagen had a greater risk of obesity, especially if their birthplaces were also rural [8].

In low and middle income countries attending university may also increase the risks of obesity [20]. Since more affluent students are most likely to attend university, higher rates of obesity are likely to be found among this group [21], especially among rural dwellers migrating to more urbanised places [22]. However, the strength of the socio-economic status (SES)–obesity relationship is likely to be context dependent. As Jin and Lu [46] have noted, with the exception of cross-national studies [6], most of the existing studies on the relationship between SES and obesity have ignored spatial variations in the nature of this relationship. This has been particularly evident in studies within particular countries where the factors producing obesogenic environments, and hence the nature and strength of the SES–obesity relationship, are likely to vary over geographic space. Thus it might be expected that more affluent students originating from higher income regions or who are currently studying in more urban and economically developed environments will be most at risk, because exposure to obesogenic factors is likely to be greater in such places [12].

While there have been numerous regional studies of obesity [10, 14, 23, 24] there have been few multi-level approaches [13, 25] which have examined the independent influence of city or regional contextual factors on obesity among young adults. The few studies which have occurred have largely focused on children and adolescents, usually at the local neighborhood level [17, 26]. While neighborhood effects are important, so too are influences which operate at other spatial scales. These may be levels of urbanization or area income differences, both of which are likely to be related to the greater availability of energy dense foods or reduced daily physical activity [11]. In addition the effects of income inequality are likely to be greater in such places and thus should strengthen the relationship between overconsumption among the rich and food insecurity among the poor [12]. Despite the importance of macro-level variables a recent review of contextual determinants of obesity paid little attention to such factors [3].

In the light of the preceding comments this paper poses two questions:

1. Independent of individual characteristics, what contextual attributes of a student’s home location are important in explaining current patterns of overweight and obesity and do these remain significant when taking into account attributes of the student’s current university city location?
2. Given the well-known link between individual SES and increased obesity in low and middle income countries, to what extent is this evident for university students and does the strength of this relationship vary across different geographic contexts?

To answer these questions the rest of the paper is organized as follows. First we outline the methodology of the study. This is followed by the results and a discussion of the most important findings, placing them in a wider context of international research on obesity and other Chinese studies. We conclude by emphasizing some of the wider theoretical and policy implications of our study.

## Methods

### Data source

This study reports data from the Global Health Professions Student Survey (GHPSS) on Tobacco Control in China GHPSS (Extended version). The GHPSS is part of the Global Tobacco Surveillance System and is a university-based survey. The GHPSS was initially completed in 31 countries between 2005 and 2007. In China the GHPSS provided a valuable source of information on health and related health behaviours, including obesity.

**Fig. 1:**
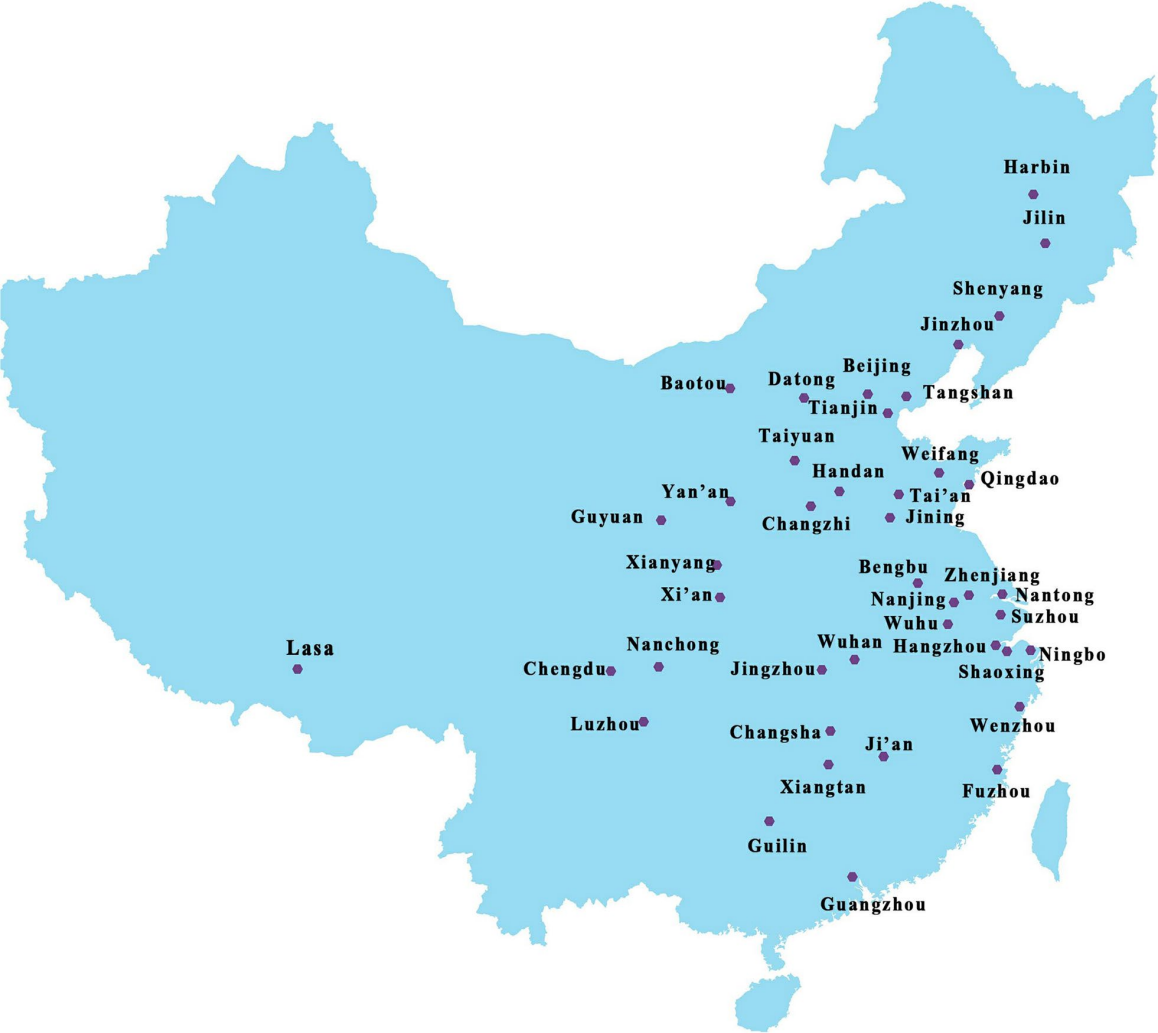
Geographical distribution of 50 universities across China, 2013

The study employed a multistage sampling design and collected the sample in 2013. In Stage 1, 50 universities with medical programs were selected from 42 cities across China and differentiated by regional location (see Fig. 1). Stage 2 of the sampling strategy involved the selection of classes within each university, and all students in these classes were eligible as the sampling frame. A more detailed description of the survey and the data can be found in Yang et al. [27]. The study was approved by the Ethics Committee at the Medical Center, Zhejiang University, and verbal consent was obtained from all participants prior to data collection.

### Measures

#### Dependent variable

Body mass index (BMI) was calculated by dividing body weight (kg) by squared height (m^2^). Overweight and obesity were defined as recommended by guidelines for the prevention and control of chronic diseases in China (Department of Disease Control and Prevention [28]): individuals with BMI scores of 24.0–27.9 kg/m^2^ were categorized as overweight, and those with scores of ≥28.0 kg/m^2^ were categorized as obese, which is the national standard [28]. As the prevalence of obesity was low in this sample of university students, the categories of overweight and obesity were combined in the analyses.

Height and weight were measured by self-report. Given the potential problems of this measure [29], we objectively measured height and weight among 170 subjects from two universities in Hangzhou in order to validate the self-reported prevalence of overweight and obesity and for obesity alone. Concordance was 97.1% for the combined prevalence of overweight and obesity, and 98.8% for obesity.

#### Individual-level independent variables

In order to control for possible individual-level confounders, questions were utilized to determine age, gender, ethnicity, parents’ occupation, monthly expenses and smoking. With few exceptions, most Chinese studies have shown that obesity tends to increase with age [30] and be higher for male children and adolescents [31, 32]. Given the well-established link between SES and obesity in China, three measures of SES, parents’ occupation, family income and monthly student expenses, were included. Occupation was recorded in three categories: operations and commercial work (operations referring to mainly farmers and workers), staff and administration (which included mainly government jobs and company management jobs); teacher, scientific and technical work) (9, 10). Family income (in RMB Yuan) was measured through the question: “how much was the income of each person in your family over the last year?” Categories ranged from less than ¥1000, ¥1000 to ¥1999, and ¥2000 and over. We also included a variable, monthly student expenses (in RMB Yuan), which was measured though the question: “how much do you spend each month?” In addition, in view of the very high rates of smoking in China, which has been related to overweight and obesity, we also included this as a background factor [13, 33–35].

#### Home location and current contextual factors

Two sets of contextual variables were included, relating to characteristics of the student’s family home location and of the university city where they were studying. In terms of the former, family location was defined in terms of both their home region (Northeast, North, Eastern, Centre, Southwest and Northwest) and whether students came from a city, county ‘town’ or rural area. In addition, gross domestic product (GDP) per capita was included to highlight differences in area income between the students’ home provinces.

Given that many studies have stressed the link between the penetration of obesogenic environments and a country’s level of urbanisation [11], we included urban population size and area income (GDP per capita) to describe the university cities where the students were studying. Finally we also determined the characteristics of the universities which the students attended. Given that different universities have different social resources and some are far more prestigious than others, then it was important to include such a measure since, because of large differences in tuition fees, university type is also an indirect measure of family income. University type was determined using the China university ranking system (“high level,” “middle level,” and “low level”) as established by the National Ministry of Education [36].

### Data analysis

All data were entered into a database using Microsoft Excel. The dataset was then imported into statistical analysis system (SAS) (9.3 version) for statistical analyses. Descriptive statistics were calculated to determine the prevalence of overweight and obesity, which was also mapped at the provincial level. For the purposes of mapping prevalence rates were defined according to the home provinces of the students in the sample.

A logistic model was utilized to assess associations between the dependent and each of the independent variables. Both unadjusted and adjusted methods were considered in the data analyses and implemented to examine these associations. SAS survey logistic procedures were applied in the unadjusted analysis, using the university as the clustering unit, in order to account for a within-clustering correlation, attributable to the complex sample for unadjusted analysis. The multilevel analysis was weighted using sampling, subject-level weights, and post-stratification weights, respectively [37].

In terms of the first study objective, we applied multilevel logistic regression models using the SAS GLIMMIX procedure (Table 2). We started with the Null Model, a three -level (individual, university, and original home province) model with random intercepts. First we constructed an individual model which included variables relating to gender, monthly expenses and smoking. The second (home location) model included the above individual factors but also three variables relating to region of origin, urban–rural background and GDP per capita of the home region. Models were run both including and excluding the student’s home region. The third (university city model) added three new contextual variables to the above individual characteristics, university type, university city GDP and city size. Finally, since we also wished to examine the relative importance of both home location and university city characteristics, the final (combined) model included all of the above variables.

It should be mentioned that family income is an appropriate indicator of family economic resources. However, family income was surveyed in only some universities and obtained for 4902 students due to a printing error in the questionnaire. Consequently we used an indirect measure of family income, monthly student expenses in the multilevel models. This was a valid measure because families tend to fund most students’ expenses. Not surprisingly the latter was significantly associated with family income (r = 0.30; p < 0.001).

With respect to the second objective, we assessed the interaction between our measure of student income, monthly expenses, and four contextual variables: the rural–urban home location of the student, university city population and the GDP per capita of the student’s home region and university city. Thus for each group we were able to assess the strength of the individual SES–obesity relationship as well as the (weighted) prevalence of overweight and obesity. We also examined the relationship between urban–rural origin and obesity by university type.

## Results

A total of 12,211 questionnaires were completed. Of those who responded 11,942 students were available for general analysis. BMI was calculated for the 11,673 respondents (97.4%) who provided complete data. There were no significant differences in demographic characteristics between responders and non-responders. Of the sample, 16% were less than 20 years of age and 21% were aged 23 years and over. There were more females (64.2%) than males, most (93%) of the students were of Han ethnicity and the majority (over 75%) came from families where the parents were engaged in operations or commercial work. Almost 38% recorded high levels of monthly expenditures (over 1500 RMB). Most students came from the countryside or townships (67%) and about half attended universities in middle-size cities.

**Fig. 2:**
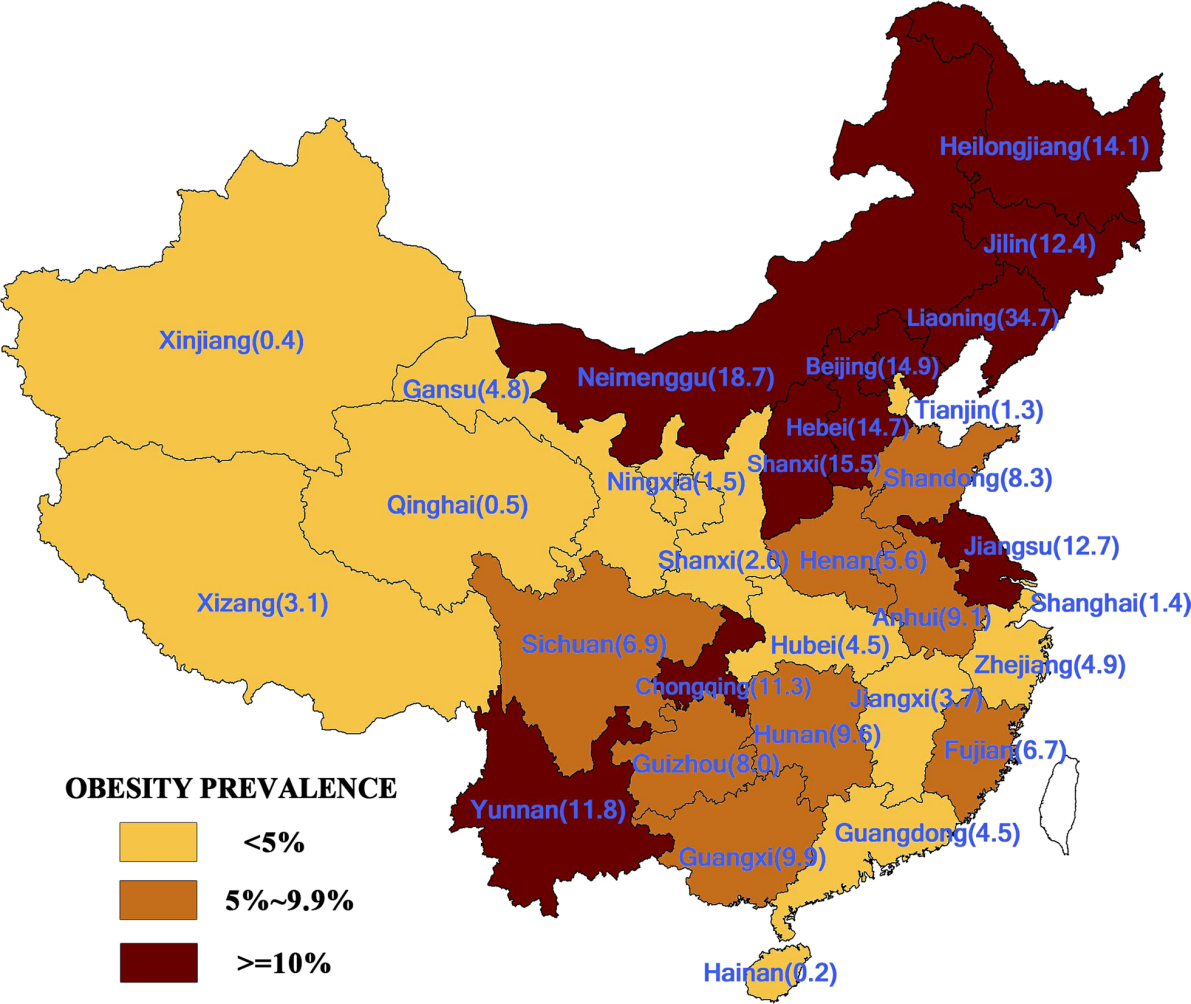
Estimated overweight and obesity prevalence of university students by their home region, 2013

Overall the prevalence of overweight and obesity in the study sample was 9.5% (95% confidence interval (CI) 7.7, 11.3%), and obesity prevalence was 2.2% (95% CI 1.3, 3.1%) there was considerable geographic variation. A higher prevalence occurred in northeast and southwest China (Fig. 2), with the highest rates being recorded in Liaoning (34.9%), Neimenggu (18.7%), Shanxi (15.5%) and Beijing (14.9%).

The unadjusted logistic analysis showed that of the individual-level variables gender (male), higher monthly expenses and smoking were associated with being classified as overweight and obese (Table 1). Parents’ occupation was not significant. However in the limited sample there was a significant relationship between family income and overweight and obesity with higher income families more likely to be overweight or obese. The unadjusted odds ratios (ORs) were 1.61** (95% CI 1.19, 2.17) (for 10,000 and over RMB vs <10,000RMB) and 1.35** (CI 1.14, 1.59) (for 20,000 and more RMB vs <10,000RMB). This relationship remained after adjusting for other individual variables [the respective ORs were 1.61** (CI 1.10. 2.20) and 1.29** (CI 1.08, 1.55)] and all variables [1.38** (CI 1.09, 1.75) and 0.82 (CI 0.57, 1.18)]. In all models there were no significant differences between monthly expenses and overweight and obesity after considering family income.

**Table 1:** Demographic characteristics of sample and overweight and obesityprevalence

Group	Unweighted N	Unweighted % of sample	Weighted % of sample	Weighted overweight and obesity prevalence	Weighted OR (95% CI)
Age (years)					
<20	1831	15.7	12.9	10.1	1.00
20	2324	19.9	31.5	6.5	0.67 (0.43, 1.07)
21	2709	23.2	30.8	7.1	0.70 (0.34, 1.41)
22	2396	20.5	14.5	8.0	0.68 (0.38, 1.21)
23 and over	2413	20.7	10.3	7.9	0.79 (0.47, 1.32)
Gender					
Male	4177	35.8	43.9	9.4	1.00
Female	7496	64.2	56.1	5.7	0.40 (0.27, 0.60)**
Ethnicity					
Han	10,884	93.2	94.6	7.6	1.00
Minority	789	6.7	5.4	6.9	0.90 (0.36, 2.26)
Father’s occupation					
Operation and commercial work	9269	79.4	71.8	9.0	1.00
Staff and administration	1681	14.4	18.8	10.3	1.66 (0.79, 1.73)
Teacher, scientific and technical work	723	6.2	9.4	11.8	1.16 (0.46, 2.97)
Mother’s occupation					
Operation and commercial work	9397	80.5	72.4	8.9	1.00
Staff and administration	1500	12.9	16.6	10.1	1.18 (0.79, 1.73)
Teacher, scientific and technical work	776	6.5	11.0	12.7	1.17 (0.45, 2.97)
Income of each person in family (RMB)					
<10,000	1773	36.2	34.0	6.1	1.00
10–19,999	1241	25.3	20.7	9.5	1.61 (1.19, 2.17)**
20,000 and over	1888	38.5	45.3	8.1	1.35 (1.14, 1.59)**
Monthly expenditures (RMB)					
<1000	1273	10.9	7.6	7.4	1.00
1000–1499	6048	51.8	49.0	8.8	1.21 (0.67, 2.16)
1500–1999	3406	29.2	30.2	8.5	1.18 (0.60, 2.30)
2000 and over	946	8.1	13.5	15.0	2.37 (1.39, 4.06)**
Academic major					
Medical	10,270	87.9	81.1	6.5	1.00
Other	1403	12.1	18.9	7.8	1.21 (0.93, 1.58)
Smoking status					
Non-smoker	10,735	92.0	87.3	8.9	1.00
Occasional smoker	706	6.1	8.9	10.7	1.22 (0.61, 2.43
Daily smoker	232	2.0	3.8	20.1	2.56 (1.33, 4.92)**
Home region					
Northeast	889	7.6	5.1	21.1	1.00
North	1785	15.2	14.4	15.3	0.67 (0.33, 1.37)
Eastern	1784	15.3	17.0	11.1	0.47 (0.24, 0.93)*
Centre	4968	42.6	39.2	7.4	0.30 (0.16, 0.55)**
Southwest	959	8.2	16.3	7.7	0.31 (0.16, 0.60)**
Northwest	1288	11.0	8.0	2.4	0.09 (0.03, 0.32)**
Urban–rural home location					
Countryside or township	3276	67.0	59.6	4.3	1.00
County town	741	15.1	17.7	9.1	2.35 (1.10, 5.02)*
City	876	17.9	22.8	15.5	5.22 (1.70, 16.3)**
GDP of home province					
<50,000	5868	50.3	51.3	8.2	1.00
50,000–99,000	3483	29.8	26.7	9.8	1.21 (0.81, 1.82)
100,000 and over	2322	19.9	22.0	12.2	1.17 (1.05, 1.41)*
Type of university					
High level	4154	35.1	58.3	11.5	1.00
Middle level	6823	53.3	39.2	6.8	0.56 (0.43, 0.74)**
Lower level	696	6.0	2.5	5.2	0.42 (0.34, 0.53)**
University city GDP (per capita)					
<50,000	3986	34.1	16.0	7.1	1.00
50,000–99,000	6221	53.3	60.7	8.9	1.28 (0.96, 1.72)
100,000 and over	1466	12.6	23.3	12.8	1.94 (1.25, 3.00)**
University city population (m)					
<1.0	3019	25.8	12.1	6.3	1.00
1.0–3.9	5866	50.2	57.7	10.1	1.68 (1.11, 2.55)*
4 and over	2788	23.9	30.2	9.8	1.62 (1.10, 2.40)*

* p < 0.05; ** p < 0.01

Of the home contextual factors, students who originated from Northeast China (Fig. 2), from larger towns and cities and from provinces with higher GDP per capita were more likely to be overweight or obese. Of the three contextual factors a more urbanised family home location increased the chances of being overweight and obese to a much greater extent than region of origin or home province GDP. Contextual characteristics of the university cities were also important. Larger and wealthier destination cities were also related to overweight/obesity prevalence levels as was the type of university. Compared to high level universities, students enrolled at lower and middle level universities had a reduced risk of being overweight or obese.

We also performed unadjusted analyses for males and females separately and some gender differences occurred (table not shown). Parental occupation became significant compared to female students who had fathers engaged in operations and commercial work, those whose fathers were teachers or were employed in scientific and technical work were more than twice as likely to be overweight or obese. The effect of daily smoking increased the risk of being overweight or obese for females (unadjusted OR 7.79; 95% CI 1.82, 33.23), but was not significant for males. By contrast, home region GDP was only significant for males (unadjusted OR 1.26; CI 1.11, 1.44), while university city GDP and population size had little effect on male trends in overweight and obesity compared to females (the respective ORs 2.39; CI 1.05, 5.48 and 1.80; CI 1.76, 2.75).

In the multilevel individual and family location models being male, a regular smoker and having higher monthly expenses remained significant (Model 1) as did the student’s urban–rural origin and area income of the home province (Model 2) (Table 2). However, home region became not significant, thus suggesting that regional differences in overweight and obesity were highly related to differences in urbanisation and levels of GDP. For this reason we re-ran Model 2 excluding home region and also omitted this variable in the final model. In the university city model no contextual variables were significant (Model 3). However, if controls were made just for individual factors then university type was significant, with students attending low level universities being less likely to be overweight or obese (OR 0.55; CI 0.40, 0.74). Similarly if controls were made just for individual factors then students attending university in the largest cities were more likely to be overweight or obese (OR 1.55; CI 1.02, 2.36). Finally in the combined model, incorporating both home and university city contextual factors, urban–rural origin, home provincial GDP and population size of the university city remained significant (Model 4).

**Table 2:** Results of multiple level model (weighted)

	Null model	Model 1 (individual model)	Model 2 (home location model)	Model 3 (university city model)	Model 4 (combined model)
Group					
Gender					
Male		1.00	1.00	1.00	1.00
Female		0.38 (0.24, 0.65)**	0.40 (0.27, 0.68)**	0.41 (0.26, 0.69)**	0.40 (0.24, 0.67)**
Monthly expenses					
<1000		1.00	1.00	1.00	1.00
1000–1499		2.32 (0.70, 7.54)	2.43 (0.72, 7.61)	1.06 (0.66, 1.76)	2.41 (0.73, 7.99)
1500–1999		1.52 (0.38, 5.86)	1.47 (0.39, 5.85)	1.03 (0.62, 1.81)	1.51 (0.42, 5.49)
2000 and over		4.07 (1.27, 13.2)**	4.10 (1.29, 13.2)**	2.65 (1.54, 3.35)**	4.23 (1.33, 13.16)*
Smoking					
Non-smoker		1.00	1.00	1.00	1.00
Occasional smoker		1.15 (0.56, 2.44)	1.19 (0.55, 2.46)	0.77 (0.37, 1.58)	1.19 (0.57, 2.49)
Daily smoker		2.12 (1.12, 3.89)*	2.07 (1.14, 3.98)*	1.30 (0.61, 2.84)	2.06 (1.06, 3.99)*
Family home location					
Rural or township			1.00		1.00
County town			2.24 (1.36, 2.75)**		2.22 (1.34, 3.61)**
City			3.79 (1.20, 12.27)*		3.73 (1.15, 12.01)*
Type of university					
High level				1.00	1.00
Middle level				0.76 (0.54, 1.15)	1.51 (0.90, 2.45)
Lower level				0.79 (0.48, 1.39)	1.14 (0.95, 1.36)
GDP per capita of home region					
<50,000			1.00		1.00
50,000–99,000			1.51 (0.89, 2.51)		1.52 (0.92, 2.54)
100,00 and over			1.43 (1.05, 1.42)*		1.43 (1.04, 1.42)*
University city GDP per capita					
<50,000				1.00	1.00
50,000–				0.92 (070, 1.21)	1.12 (0.55, 2.29)
100,000–				1.21 (0.66, 1.21)	1.39 (0.55, 3.71)
City population (million)					
<1.0				1.00	1.00
1–3.9				1.28 (0.82, 1.93)	1.71 (1.10, 2.68)*
4 and over				1.41 (0.80, 2.35)	1.48 (1.04, 2.31)*
Fixed parameters	37.36 **	19.12**	17.25**	16.42**	15.75**
Random parameters between universities	4.41**	3.89**	4.25**	2.43*	2.25*
Random parameters between the original provinces	62.04**	39.87**	35.41**	37.82**	15.89**

* p < 0.05; ** p < 0.01

Controlling for gender, monthly expenses and smoking, students who originated from cities versus rural and township areas were between 60 and 82% more likely to be overweight or obese. In addition we investigated the interaction between home location and individual level expenses since both factors have independent influences on obesity. However, analyses showed no significant interaction between these two factors (Wald Chi Square: 2.74, p: 0.0979).

In terms of the relationship between student monthly expenses and obesity and urban/area income contextual factors the results suggest that association between individual SES and obesity was strongest for students who originated from rural areas and for those who attended universities in smaller cities (Table 3). The results are less clear for home region GDP but in the case of university city GDP higher income students were more likely to be overweight or obese in poorer cities. In terms of the actual prevalence of obesity the highest rates occurred among more affluent students originating from (28.7%) or currently living in (21.2%) larger places and among more affluent students coming from wealthier areas.

**Table 3:** Relationships between student monthly expenses and obesity by urban–rural home location and area income

Monthly expenses	Overweight and obesity prevalence (%)	Adjusted OR (95% CI)^a^	Overweight and obesity prevalence (%)	Adjusted OR (95% CI)^a^	Overweight and obesity prevalence (%)	Adjusted OR (95% CI)^a^	Overweight and obesity prevalence (%)	Adjusted OR (95% CI)^a^
	Home location of student		University city population		Home region GDP per capita		University city GDP per capita	
	Rural/township		< 1 million		Low		Low	
<1000	1.0	1.00	1.5	1.00	4.3	1.00	5.5	1.00
1000–1499	4.3	1.40 (0.64, 3.10)	5.9	1.73 (1.06, 2.83)*	9.2	1.34 (0.88, 2.06)	11.1	1.13 (0.69, 1.86)
1500–1999	4.3	1.42 (0.65, 3.18)	7.9	2.03 (1.49, 2.77)**	7.0	1.96 (1.34, 2.88)**	14.6	1.54 (1.03, 2.29)*
2000 and over	8.5	2.46 (1.22, 4.68)*	8.4	2.33 (1.17, 4.68)**	10.0	1.88 (1.15, 3.07)*	14.4	2.26 (1.45, 3.53)**

	Small city		1.0–3.9 million		Medium		Medium	
<1000	0.0	N/A	5.6	1.00	11.9	1.00	8.7	1.00
1000–1499	10.3	N/A	9.6	1.04 (0.71, 1.51)	8.4	0.93 (0.70, 1.24)	8.9	0.90 (0.59, 1.38)
1500–1999	11.8	N/A	10.5	1.27 (0.89, 1.78)	9.5	1.07 (0.77, 1.47)	6.0	1.05 (0.73, 2.00)
2000 and over	1.3	N/A	13.4	1.59 (1.08, 2.35)*	20.2	1.27 (0.87, 1.87)	17.7	1.35 (0.92, 2.00)

	Large city		4 million and over		High		High	
<1000	23.6	1.00	13.4	1.00	4.2	1.00	5.5	1.00
1000–1499	19.3	0.61 (0.20, 1.87)	8.1	0.61 (0.41, 0.92)*	7.6	2.67 (1.50, 4.77)**	6.3	0.62 (0.40, 1.02)
1500–1999	3.4	0.60 (0.25, 1.48)	5.9	0.75 (0.56, 1.04)	10.4	3.01 (1.83, 4.94)**	10.4	0.98 (0.62, 1.56)
2000 and over	28.6	1.51 (0.54, 4.18)	21.2	1.15 (0.76, 1.47)	18.5	1.93 (1.14, 3.28)*	10.5	1.08 (0.59, 1.81)

Adjusted odds ratios in italics are statistically significant at the * p < 0.05; ** p < 0.01

N/A = sample sizes too small

^a^Controlling for gender

## Discussion

This aim of this study was twofold; to assess the importance of home location and university contextual effects on patterns of student overweight and obesity and to investigate the extent to which the SES–obesity relationship varied depending upon the geographic context. While this is not the first Chinese study to investigate obesity among young adults [9] or university students in particular [21], it is the first Chinese research to examine these factors using a multi-level framework taking into account both past and current locations. Internationally, the research is also one of the few studies [46, 47] to consider variations in the nature of the SES–obesity relationship within a particular national context.

With respect to the first objective three main conclusions are evident. First, with respect to the student’s home environment, independent of individual characteristics, levels of urbanization and provincial GDP per capita emerged as the key predictors of overweight and obesity. Students who came from county towns or larger cities were twice to almost four times more likely to be obese or overweight compared to students originating from rural areas. Similarly those who were born in more affluent regions were more likely to be overweight or obese, independent of their own individual income status. These patterns suggest the importance of lifestyle and dietary factors on overweight and obesity because students’ basic lifestyles are partly formed during childhood and adolescence. These findings are similar to other research both in China [21] and elsewhere [13, 18, 26] which points to the significance of early life conditions on patterns of adult obesity.

Second, there was also evidence of contextual influences in the destination cities of the students. Students who attended university in the larger cities were more likely to be overweight or obese compared to students who lived in smaller cities and this effect remained significant in the final model after controlling for the urban–rural home origins of students. These results are similar to the findings of Ji and Chen [38] who found that over the period 1985–2010 the rate of increase in overweight and obesity (amongst children and adolescents) was greatest in the largest cities, the authors suggesting that adult prevalence rates in such places were approaching those found in developed countries. They also support the results of other studies of students [21] and adults [39] as well as children and adolescents in China [38] where urban–rural differences in obesity, despite decreasing in recent years [40], are still very apparent.

Although in the expected direction, university city GDP was not significant. University type was significant when just individual factors were controlled for but not in the final model nor in the university model when the two other university contextual characteristics were included. This most likely reflects the fact that the most prestigious universities are located in the largest cities, places where students will be most exposed to obesogenic environments. Nevertheless the pressures of studying at China’s most prestigious universities should also be taken into account. One can only speculate that sedentary behavior is more likely to be common among such students who have little time for other activities. Li et al. [21], for example, reported that obese students in Guangdong were more likely to indicate that they never exercised or engaged in daily exercise. Thus it was no surprise that, when university type was considered, the highest prevalence of overweight and obesity was typical of students with urban origins studying at China’s top universities.

Third, the findings suggest that characteristics of the home locations of students are more important than those of the places to which they migrated in influencing overweight and obesity. This should not be surprising given the short length of residence of many of the students in the university cities. It would be tempting to suggest that the selective migration of more affluent students from their homes to university accounted for most of the variation in obesity amongst this student sample. However, other studies have suggested that differences in the prevalence of obesity could not be accounted for by birthplace or later selective migration [8] and that resident children are more likely to be obese than migrant children [32]. Such findings thus point to the importance of local context effects, which are likely to become more important over time, in affecting obesity prevalence among the student population. Thus the fact that urban size remained significant in the final model suggests the importance of destination factors in modifying patterns of obesity amongst the most at-risk populations.

**Fig. 3:**
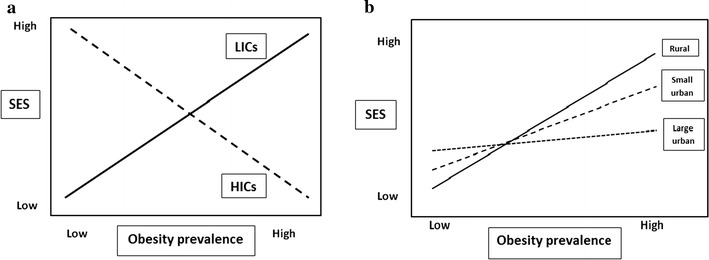
Estimated relationships between socio-economic status and obesity between high and low income countries and by levels of urbanisation within low/middle income countries. **a** Between country differences, **b** within country differences

With respect to the second objective, the study found further evidence of the well known link between individual socio-economic status and obesity, which was evident in all the models. Unlike western countries where obesity is highest amongst the poor and certain ethnic groups [24], the pattern of higher prevalence of obesity among students from higher income families is typical of countries in the earlier stages of the nutrition transition [5, 6]. However, the fact that income differences in obesity were generally strongest for students originating from rural locations and for those who attended university in smaller cities suggests that the nutrition transition is at an earlier stage than in larger cities where, although obesity rates are higher, individual SES differences are much less pronounced (Fig. 3b). Thus the results suggest a need for more thought to be given to geographical variation in the SES–obesity relationship especially in countries as diverse as China. In other words, models of the nutrition revolution, rather than focusing just on international differences in the relationship between SES differences and obesity (Fig. 3a), need to take greater account of the forces operating within low and middle income countries. For example, at what point in the urbanization process do factors relating to obesogenic environments start to become much more important compared to individual-level determinants of obesity? Do particular thresholds exist? Such considerations are important in all low and middle income countries where the obesogenic epidemic is at an earlier stage. Answering such questions obviously has implications for population programs aiming to target the most at-risk groups.

Thus, in contrast to some other views [10] the geographic distribution of obesity cannot simply be read off from individual SES variations, but also reflects a range of context effects which will modify the individual SES–obesity relationship. Important here is the level of urbanization and regional affluence which will be highly correlated with the penetration of obesogenic environments. As He et al. [12] have suggested, these will include the greater availability of energy dense foods at a cheaper cost, the spread of obesity-related health knowledge [13] or the adoption of higher-SES groups of western cultural norms regarding body shape, all of which may narrow SES differences in obesity. On the other hand, higher rates of income inequality and increased food consumption among the rich [41, 42] or the effects of transportation infrastructure and other labour saving devices which result in reduced daily physical activity [11], are likely to increase social disparities in obesity. The above results thus suggest an urban–rural diffusion of the obesity epidemic in China. However, while the spread of the obesity epidemic to rural areas, to some extent, has already been identified [30, 38, 47] exactly how the local environment helps shape the social distribution of obesity in different places remains unclear. Thus more research which focuses on the role of contextual factors influencing obesity at smaller spatial scales, such as cities and neighbourhoods, would seem to be a high priority.

### Limitations

This study has a number of limitations. First, and most important, is that our range of contextual factors was relatively limited. Although it was beyond the scope of this study greater attention to cultural factors affecting urban and regional differences in diet, the food environment and to the effects of income inequality on patterns of food consumption is necessary. More attention also needs to be paid to the nature of obesogenic environmental factors and they affect different SES groups. Second, the study was based on cross sectional design, which precludes causal inference and calls for cohort and other longitudinal studies on overweight and obesity in China to advance etiologic understanding, and to inform the design and evaluation of generic and tailored interventions. Third, the sampling frame was university students, most of which were medical students. Hence, the findings are not generalizable to the whole of China. Fourth, because not all universities recorded data for the urban–rural origins of the students, this was available for only 41.9% of the total sample. As a result the sample appears to under represent urban students (those who came from cities and county towns).

## Conclusion

This study adds important insights about the impact of home and university environments on the prevalence of overweight and obesity among university students. By emphasizing the importance of contextual effects we suggest that more attention needs to be paid to the environmental conditions, such as urbanization and rapid economic development. These have helped change health beliefs, patterns of food consumption, health behavior and lifestyles and have contributed to the growing obesity epidemic in China and other low and middle income nations. Many of these factors have influenced the growing obesity of children and adolescents, but, as would be expected, have become particularly apparent among young adults. As Dutton and McLaren [43] have suggested modeling these population-level factors is an important avenue for future research. Given that not all places or population groups are equally affected then more attention to how local environments are changing and the effects of such changes on the diet, lifestyles, as well as the social norms and perceptions of obesityon the part of different groups is necessary.

Effective strategies that take into account contextual influences are also needed to implement policy and public health interventions to prevent overweight and obesity, especially since that there is minimal evidence that the obesity epidemic is slowing down. However, central and provincial governments in China have largely ignored food and other policies which may go some way towards slowing the growth of obesity [44] although the recent policy shift encouraging more healthy cities suggests that this view is changing [45]. This is also true among university environments and thus this study provides preliminary evidence for public health policymakers and educators and suggests the need for an approach for intervening to avert or reduce overweight and obesity among college students. Such an approach needs to take account of the diverse origins of students, since such differences will be important in affecting the social distribution of obesity and the attitudes of students to any policy interventions. Thus we recommend that there is a need for strategies which address both environmental and individual factors as such a multi-faceted approach is essential to help curb the growing obesity epidemic.

## Abbreviations

GDP: gross domestic product
SES: socio-economic status
GHPSS: Global Health Professions Student Survey
BMI: body mass index
SAS: statistical analysis system
